# Polarimetric Stationarity Omnibus Test (PSOT) for Selecting Persistent Scatterer Candidates with Quad-Polarimetric SAR Datasets

**DOI:** 10.3390/s20061555

**Published:** 2020-03-11

**Authors:** Xingjun Luo, Changcheng Wang, Peng Shen

**Affiliations:** 1 School of Geosciences and Info-Physics, Central South University, Changsha 410083, China; luoxingjun@csu.edu.cn (X.L.); shen-peng@csu.edu.cn (P.S.); 2 Key Laboratory of Metallogenic Prediction of Nonferrous Metals and Geological Environment Monitoring Ministry of Education, Changsha 410083, China; 3 Hunan Key Laboratory of Nonferrous Resources and Geological Hazards Exploration, Changsha 410083, China

**Keywords:** persistent scatterers, polarimetric optimization, deformation monitoring

## Abstract

In the traditional single polarimetric persistent scatterers interferometric (PSI) technology, the amplitude dispersion index (ADI) is usually used to select persistent scatterer candidates (PSC). Obviously, based on single polarimetric information, it is difficult to use the statistical characteristics for comprehensively describing the temporal stability of scatterers, which leads to a decrease in persistent scatterer (PS) density. Considering that the temporal polarimetric stationarity of PS, the paper is based on complex Wishart distribution and proposes the polarimetric stationarity omnibus test (PSOT) for identifying PSC. The nonstationary pixels can be removed by the preset significance threshold, which reduces the subsequent processing error and the calculation cost. Then, the exhaustive search polarimetric optimization (ESPO) method is selected for improving the phase quality of PSCs while suppressing the sidelobe of the strong scatterer effectively. For validating the effectiveness of the proposed method, we select a time-series quad-polarimetric ALOS PALSAR-1 images in an urban area as experimental data and mainly perform five group experiments for detailed analysis, including the PSOT+ESPO, ADI+ESPO, ADI+HH, ADI+HV, and ADI+VV. The results show that the proposed PSOT+ESPO method has a better performance on both PSC selection and interferometric phase optimization aspects than that of other methods. Specifically, compared to the last four methods, both the PSCs and PSs identified by the proposed PSOT+ESPO are more concentrated in the high-coherence region. The PSs with the standard deviation (STD) less than 5mm in the PSOT+ESPO method account for 94% of all PSs, which is greater than that of the ADI+ESPO, ADI+HH, ADI+HV, and ADI+VV methods, respectively.

## 1. Introduction

Interferometric synthetic aperture radar (InSAR) technology is one of the most popular geodetic techniques with the advantages of high precision, high resolution and all-weather work. Since Gabriel first used differential InSAR (DInSAR) to obtain deformation information of farmland in 1989 [1], researchers have successively improved DInSAR technology, such as improvement of the interferometric phase, and separation of multiple-phase signals, including orbital, atmospheric and residual topographic phases [2,3,4,5], etc. However, the accuracy of DInSAR is still affected by factors such as temporal and spatial decorrelation and atmosphere delay. 

Therefore, to overcome the mentioned problems above, Time-series InSAR (TS-InSAR) technology based on DInSAR technology has gradually developed [6] and mainly includes the persistent scatterers interferometric (PSI) [7] and the small baseline approaches (SBAS) [8]. The PSI can identify the targets, persistent scatterers (PS), with stable scattering characteristics on the ground and monitor the surface deformation based on the reliable phase and amplitude information. PSI is widely used in volcanoes, earthquakes, urban subsidence, and landslides [9,10,11,12,13,14]. Especially in urban areas, artificial buildings in urban areas can be considered as ideal persistent scatterers (PS) [15], and the PSI technology is based on the PSs to explore the impact of human or natural activities on cities [16]. 

However, how to identify PS pixels more steadily is still a widely concerned problem, and it restricts the application of the PSI technology. More reliable PSs mean that the noise can be suppressed more effectively, and the accuracy of deformation solution can be improved in the subsequent processing. Some scholars have done some work in improving PS density. Shanker et al. proposed a method of maximum likelihood ratio to find more PS, and the average phases of PS pixels clearly show the slip along the Hayward fault [17]. Foroughnia et al. proposed a novel iterative PSI method (IPSI) to increase the PS points, which are lost in the PS-InSAR technique due to unwrapping failure [18]. Xiang et al. fully take advantage of the signal amplitude and phase information in the monitored scene and propose a combined PS selection (CPSS) method [19]. Gheorghe et al. Combine the ascending and descending SAR images to improve the monitoring density [20]. With the development of tomography SAR technology, Budillon et al. address the complementarity of the two techniques, and in particular it assesses the increase of measurement density that can be achieved by adding the double scatterers from SAR tomography to the persistent scatterer interferometry measurements [21]. These methods mentioned above are based on single-pol image for the improvement and application of PSI technology. 

With the number of multi-polarization satellites increasing, it is necessary to introduce polarimetric observation for more effectively selecting PS and improving phase quality [22,23,24,25,26]. Polarimetric optimization becomes a direction of using polarimetric images to monitor deformation. The dual-polarimetric SAR dataset supported by Sentinel-1A and TerraSAR-X has been proved to be able to obtain more PSs with higher coherence [22,27,28]. It has been demonstrated that the polarimetric information introduction can increase the PS density. Higher PS density can help to detect more local deformation information and construct a more robust unwrapping network for removing the atmospheric phase [29]. To obtain PSs with stable phase, persistent scatterer candidates (PSC) need to be extracted firstly from full scenes. The effective identification of PSC efficiently extracts most high-coherence pixels while reducing the subsequent processing error and the calculation cost. Usually, the traditional PSI technology uses amplitude dispersion index (ADI) as the quality indicator to identify PSC [30]. However, in traditional PSI technology, the single-pol statistical characteristics are not comprehensively used for describing the temporal stationarity of scatterers. 

In addition, in the urban area, the side lobes of high-intensity scatterers will interfere with nearby scatterers and even cover up low-intensity scatterers [31]. These problems are challenges when using PSI technology. In [24,32,33], Navarro-Sanchez et al. propose a general framework for PSI technology based on the exhaustive search polarimetric optimization (ESPO) algorithm [34]. The ESPO method optimizes the phase quality while suppressing noise and sidelobe, and allows some scatterers that are only sensitive to specific polarizations to be detected. However, the computational cost of the ESPO algorithm increases exponentially with the improvement of accuracy, which also limits the use of this method. 

In this paper, a PSC selection method based on quad-polarimetric SAR image is proposed. Considering the temporal stationarity of PS, the paper is based on complex Wishart distribution and proposes the polarimetric stationarity omnibus test (PSOT) for identifying PSCs [35]. The proposed PSOT method can greatly reduce the amount of calculations of the ESPO algorithm and increase the number of PSs. The selected PSCs are optimized by ESPO, and it can improve phase quality and reduce noise and sidelobe. Finally, deformation velocity can be estimated by the PSI technology. The hypothesis and method are verified on real SAR images, and all experimental results are evaluated quantitatively.

The rest of this article is as follows. Section 2 presents the basic theory and flowchart of the proposed method. Section 3 describes the study area and the polarimetric SAR datasets for experiments. Section 4 is the experimental results and detailed analysis. Finally, Section 5 gives the conclusion. 

## 2. Method

### 2.1. Persistent Scatterer Candidates Identification Based on Polarimetric Stationarity Omnibus Test (PSOT)

In an ideal situation, for a stable scatterer on the ground, the polarimetric information in time-series remains unchanged. In this paper, a time-series Polarimetric Stationarity Omnibus Test (PSOT) is proposed to evaluate whether the polarimetric information of scatterers changes in time. For the quad-polarimetric sensor, the scattering matrix S can be obtained:

(1) $$S=\left[\begin{array}{c} S_{HH}\;\;S_{HV}\\ S_{VH}\;\;S_{VV} \end{array}\right]$$

In a monostatic radar system, the scattering target satisfies the reciprocity and the Sinclair matrix is restricted to a symmetric matrix, i.e., $S_{HV}=S_{VH}$. Therefore, 3-dimension Pauli feature vector $\underline{k}$ is:

(2) $$\underline{k}=\frac{1}{\sqrt{2}}{\left[S_{HH}+S_{VV}\;S_{HH}-S_{VV}\;S_{HV}+S_{VH}\right]}^{T}$$

The polarimetric coherence matrix T can be obtained by the cross product between $\underline{k}$ and its conjugate transpose [34]

(3) $$T={\underline{k}}_{i}\cdot {{\underline{k}}_{i}}^{†}$$

where ${\;}^{†}$ denotes the conjugate transpose. 

We supposed that there are k quad-polarimetric observations in time-series, let $\Sigma_{i}=T_{3}^{i}$, (2≤i≤k), and the $X_{i}=\;n{\hat{\Sigma }}_{i}\;$. The $\Sigma_{i}$ (and the $X_{i}$) are p by p (p = 3 for quad-polarization image), following the complex Wishart distribution, i.e.,$\;X_{i}\;\sim \;W_{T}\left(p,n,\Sigma_{i}\right)$. Further, $X=\;\sum_{i=1}^{k}X_{i}\sim W_{T}\left(p,nk,\;\Sigma \right)$. 

It is supposed that the polarimetric information of the ground scatterers in *k* observations is unchanged, i.e., the polarimetric stationarity hypothesis. The complex coherency matrix is stationary under the polarimetric stationarity hypothesis. In order to evaluate whether all the complex covariance matrixes are equal when k≥2, the null hypothesis is tested [22]:

(4) $$H_{0}:\Sigma_{1}=\Sigma_{2}=\;\cdots \;=\Sigma_{k}$$

For all cases, the statistic Q can be constructed:

(5) $$Q={\left\{k^{3k}\frac{\prod_{i=1}^{k}\left|X_{i}\right|}{{\left|X\right|}^{k}}\right\}}^{n}$$

where | · | denotes the determinant of the matrix. If the hypothesis is true (“under H0” in statistical parlance), Q=1 and it means the polarimetric stationarity.

For the logarithm of the test statistic we get [36]:

(6) $$\ln Q=n\left\{3k\ln k+\;\overset{k}{\underset{i=1}{\sum }}\ln\left|X_{i}\right|-k\ln\left|X\right|\right\}$$

Setting

(7) $$\{\begin{array}{c} f=9\left(k-1\right)\\ \rho =1-\frac{17}{18\left(k-1\right)}\left(\frac{k}{n}-\frac{1}{nk}\right)\\ \omega_{2}=\frac{3}{\rho^{2}}\left(\frac{k}{n^{2}}-\frac{1}{{\left(nk\right)}^{2}}\right)-\frac{9\left(k-1\right)}{4}{\left(1-\frac{1}{\rho }\right)}^{2} \end{array}$$

The probability of finding a smaller value of −2ρlnQ is $\left(z=-2\rho \ln q_{obs}\right)$

(8) $$P\left\{-2\rho \ln Q\leq z\right\}≅P\left\{\chi^{2}\left(f\right)\leq z\right\}+\omega_{2}\left[P\left\{\chi^{2}\left(f+4\right)\leq z\right\}-P\left\{\chi^{2}\left(f\right)\leq z\right\}\right]$$

$P\left\{-2\rho \ln Q\leq -2\rho \ln q_{obs}\right\}=P\left\{Q\geq q_{obs}\right\}$ is the change probability, $1-P\left\{-2\rho \ln Q\leq -2\rho \ln q_{obs}\right\}=P\{Q<q_{obs}\}$ is the no-change probability [37,38]. 

It is worth noting that when the number of looks of the original image is smaller than the matrix dimension, the coherency matrix is singular and no longer obeys the complex Wishart distribution. In order to maintain the spatial resolution of the original image and the accuracy of deformation extraction, a direct way to solve this problem is to adjust the non-diagonal elements for forcing the polarimetric coherence matrix to be a full rank matrix [39,40]. Therefore, the forced polarimetric coherence matrix $T^{\prime }$ can be expressed as:

(9) $$\{\begin{array}{c} \forall i=j,\;T_{i,j}^{\prime }=T_{i,j}\;\;\;\;\;\;\;\;\;\;\;\;\;\;\;\;\;\;\;\;\;\;\;\;\;\;\;\;\;\;\;\;\;\;\;\;\;\;\;\;\\ \forall i\neq j,\;T_{i,j}^{\prime }=cT_{i,j},\;c=\sqrt[{3}]{\min\left(n/q,1\right)} \end{array}$$

where $T_{i,j}$ represents the elements of row i and column j in the original coherence matrix; $T_{i,j}^{\prime }$ represents the elements of the forced coherency matrix; n is the Equivalent Number of Looks (ENL); q is the dimension of matrix T; q=3 for the quad-polarimetric image. When the forced coherence matrix is full rank, the equivalent number of looks of the corresponding data n is considered to be 3, which is equal to the matrix dimension.

### 2.2. Polarimetric Optimization of PSC Using Exhaustive Search Polarimetric Optimization Method

Usually, traditional single-pol PSI technology is based on a co-polarized polarization (i.e., HH or VV) image to monitor the ground deformation, because the image quality in co-polarized polarization is better than that in cross-polarized polarization (i.e., HV). With the support of quad-polarimetric SAR images, it is possible to find an optimal polarization in the quad-polarimetric signal space [32], whose phase quality is better than that of the co-polarized polarization (i.e., HH or VV) image. Navarro-Sanchez et al. proposes the ESPO algorithm for polarimetric optimization in PSI technology, which can find the optimal polarization to improve phase quality [32].

The quad-polarimetric observation information of the scattering target can be denoted by the complex scattering vector $\underline{k}$. In order to obtain the interferometric phase and coherence of quad-polarimetric images, we need to convert $\underline{k}$ to μ using unitary complex vector ω [23]:

(10) $$\mu =\omega^{†}\underline{k}$$

where † denotes the conjugate transpose; μ denotes a scalar complex scattering coefficient, which is equal to single look complex (SLC) image. Therefore, we can apply the existing PSI techniques to μ. For quad-polarimetric image, ω can be parameterized by the four parameters of ω(α,β,δ,ψ), which depend on the geometry and electromagnetic properties of the scatterers:

(11) $$\omega =\left[\begin{array}{c} \cos\left(\alpha \right)\\ \sin\left(\alpha \right)\cos\left(\beta \right)e^{j\delta }\\ \sin\left(\alpha \right)sin\left(\beta \right)e^{j\psi } \end{array}\right],\{\begin{array}{c} 0\leq \alpha \leq \pi /2\\ 0\leq \beta \leq \pi /2\\ -\pi \leq \delta <\pi \\ -\pi \leq \psi <\pi \end{array}$$

The PSI technology generally uses amplitude dispersion index (ADI) $D_{a}$ to identify PSC. In this regard, the proposed ESPO method purposefully takes the minimum $D_{a}$ of the pixel in the time-series as the goal of polarimetric optimization. $D_{a}$ can be expressed as [22]:

(12) $$D_{a}=\frac{\sigma_{\alpha }}{\bar{\alpha }}=\frac{1}{\bar{\left|\omega^{†}\underline{k}\right|}\sqrt{k-1}}\sqrt{\overset{k}{\underset{i=1}{\sum }}{\left(\left|\omega^{†}{\underline{k}}_{i}\right|-\bar{\left|\omega^{†}\underline{k}\right|}\right)}^{2}}$$

where k denotes the number of images, the upper line denotes the average value, and |∗| denotes the absolute value. In the PSC selection process of PSI, a pixel whose amplitude deviation is less than the preset threshold can be selected as a PSC. Here we use the exhaustive search polarimetric optimization (ESPO) algorithm to search for the ω to minimize $D_{a}$ [33].

**Figure 1:**
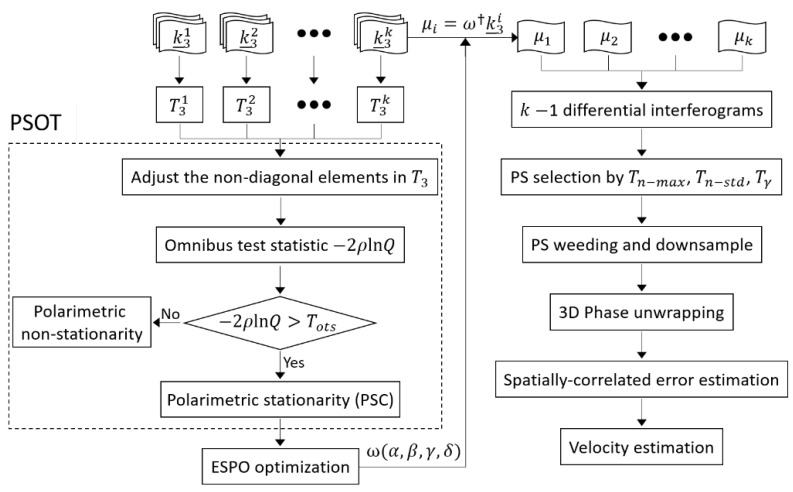
The flowchart of the proposed method. $T_{ots}$: significance threshold of polarimetric stationarity hypothesis test; $T_{n-max}$: threshold for the maximum noise allowed for a pixel; $T_{n-std}$: threshold for noise standard deviation; $T_{\gamma }$: threshold for temporal coherence.

Figure 1 is the algorithm flow of this paper. The quad-polarimetric image obtained by the satellite can be denoted as scattering vector $\underline{k}$. The complex coherency matrix T, which contains all polarimetric scattering information of ground object, can be obtained by the cross product between $\underline{k}$ and its conjugate transpose. Then the significance level of each pixel is computed with the proposed PSOT method. Next, the PSCs obeying the polarimetric stationarity hypothesis are selected by setting the threshold $T_{ots}$. The optimal SLC image μ can be obtained by the ESPO method, and then k−1 differential interferograms can be obtained. In this paper, StaMPS technology is used for the subsequent processing of PSI on the selected PSCs. PS can be selected by controlling the threshold values (i.e., $T_{n-max}$, $T_{n-std}$ and $T_{\gamma }$), and downsampling are carried out. Spatial-correlation errors (i.e., DEM, atmosphere and orbit error) are estimated and removed after 3D phase unwrapping. Finally, the deformation velocity can be estimated.

## 3. Datasets

**Figure 2:**
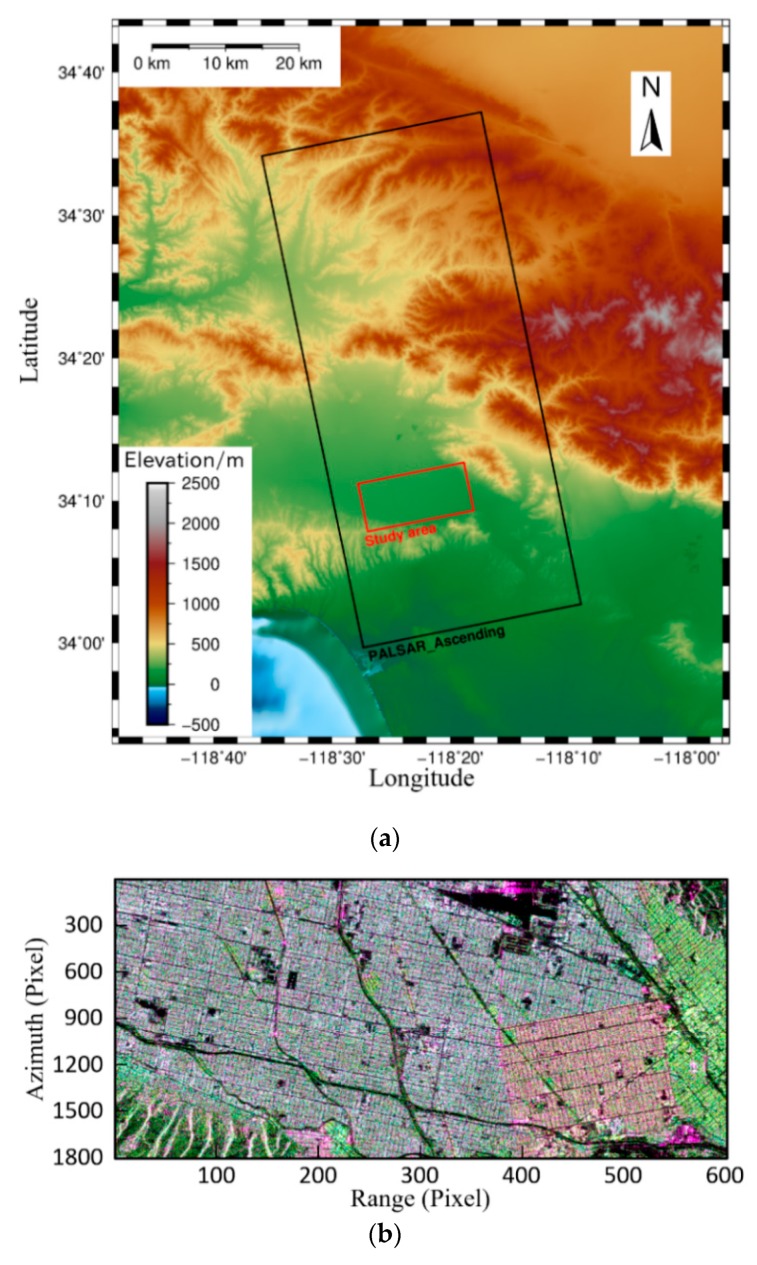
The scope and scattering characteristics of the study area. (**a**) Scope of the synthetic aperture radar (SAR) image: The black rectangle shows the spatial range of the original image, and the red rectangle shows study area; (**b**) Composite RGB image of the study area—red: ${\left|S_{HH}-S_{VV}\right|}^{2}$, green:$\;{\left|S_{HV}+S_{VH}\right|}^{2}$, blue: ${\left|S_{HH}+S_{VV}\right|}^{2}$.

In order to verify the effectiveness of the proposed method, 13 scene quad-polarimetric ALOS PALSAR-1 images covering the San Fernando Valley CA are used. The coverage of the image is shown in Figure 2a, the black rectangle shows the spatial area of the original image, and the red rectangle shows the study area. This paper mainly selects the urban area as the study area, and the main scattering mechanism is double-bounce scattering (Figure 2b).

The quad-polarimetric PALSAR-1 datasets acquisition time is between June 8, 2006, and August 1, 2009. The temporal and spatial baselines of the dataset are listed in Table 1. The image of April 26, 2007 is selected as the master image. The estimated ENL of the original image is 0.7296.

## 4. Discussion

### 4.1. Selection of Significance Threshold

**Figure 3:**
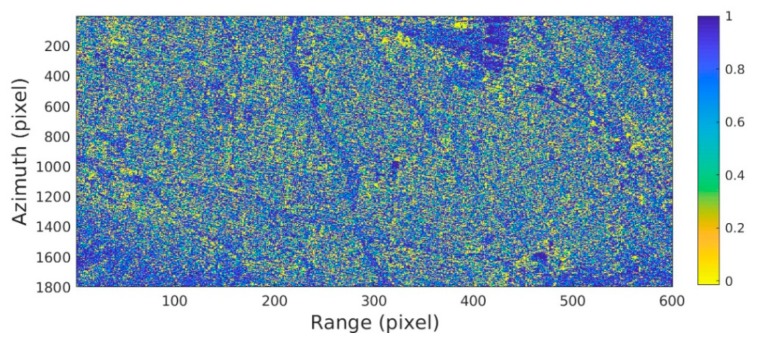
Significance level of polarimetric stationarity in the experimental area.

By evaluating the polarimetric stationarity of the pixels, the significance level of the hypothesis test of all the pixels in the study area is obtained in Figure 3. At airports, highways, vegetated areas, and the foreshortening area, the possibility of maintaining stable polarimetric characteristics is relatively low (high significance). The nonstationarity of the airport and highway is caused by the randomness and weakness of the echo signal. The vegetation growth and volume scattering variation make the vegetation area instable. The nonstationarity of the foreshortening area is caused by the backscatter signal aliasing.

Except for the regions with a significantly higher significance level (significance level >0.6), the remaining areas maintained a certain degree of stable polarimetric characteristics (significance level ≤0.4). The main surface cover types of these areas are urban buildings, and the main scattering mechanism is double-bounce scattering. Different significance thresholds are used to identify PSC, and the number and statistical characteristics of selected PSCs (and PSs) are also different. We conducted further experiments using HH polarization for identifying PSC, and then the PSC was post-processed using StaMPS to obtain PS. In StaMPS, the noise pixel can be removed by the downsampling parameter (merge_resample_size), but at the same time, the number of PS pixels will inevitably be reduced. The resolution of azimuth and ground range direction of the experimental data used in this paper is 3.54 m and 24.1 m, respectively. Therefore, we set the downsampling parameter to 25 m.

**Figure 4:**
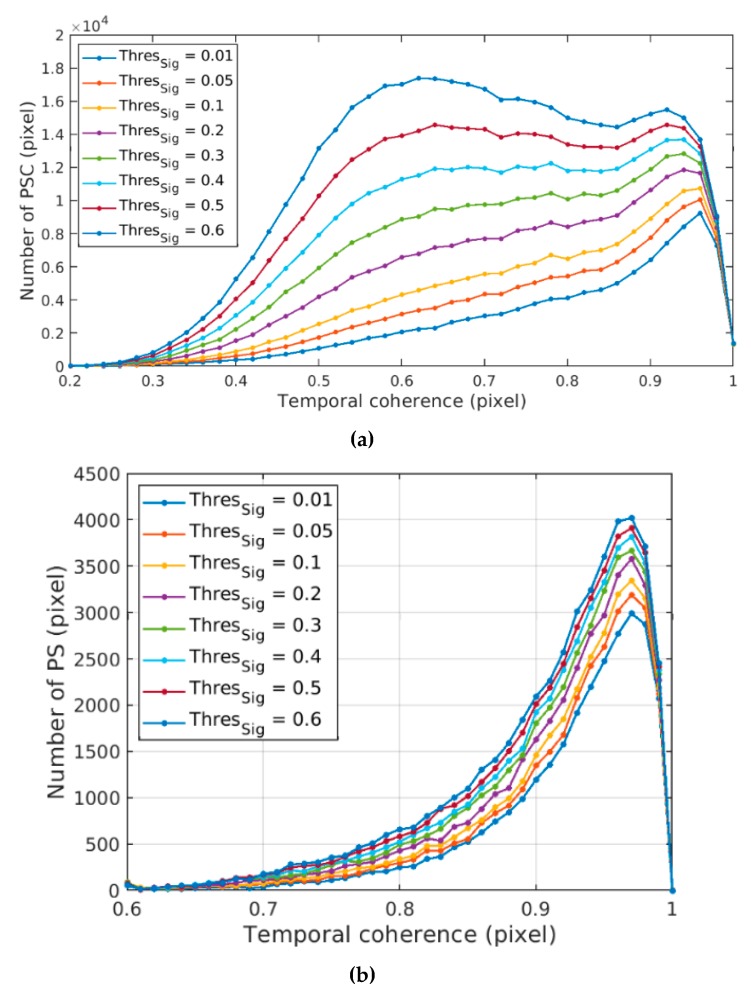
Temporal coherence distribution of persistent scatterer candidates (PSCs) and persistent scatterers (PSs) in HH polarization under different significance thresholds. (**a**) PSC; (**b**) PS.

For studying the influence of increased significance threshold on the temporal coherence distribution, different significance (≤0.6) are selected for the PSC selection experiment, and the corresponding temporal coherence distribution of PSC and PS in HH polarization are obtained. Figure 4a shows that with the decrease of significance threshold, PSC can be greatly reduced. Pixels in the low-coherence range (< 0.85) are more sensitive to the change of significance threshold, which also shows that the PSOT can extract PSC effectively. In Figure 4b, the decrease of significance threshold mainly affects PS with temporal coherence between 0.6–1. The change of significance threshold has little effect on the number of PS. A higher threshold can extract more PS. Even if the significance threshold is set to 0.01, which is very strict, the number of PS (HH polarization) can still stay at 28355.

**Figure 5:**
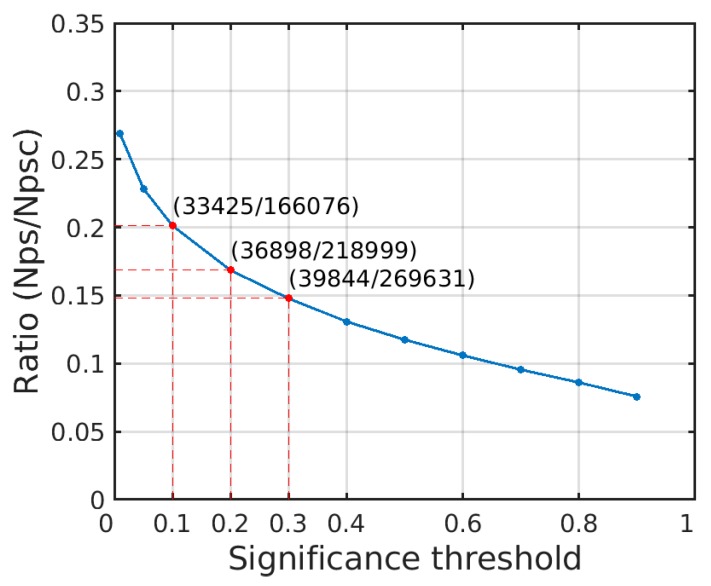
Changes of the ratio of Nps to Npsc under different significance thresholds. Nps: the number of PS, Npsc: the number of PSC. The tags in the figure correspond to the number of PS and PSC under different significance thresholds, namely (Nps/Npsc).

In Figure 5, within the range of significance threshold greater than 0.3, the ratio of Nps (the number of PS) to Npsc (the number of PSC) are approximately linear. Therefore, it can be considered that the pixels above the significance threshold of 0.3 have no significant polarimetric stationarity characteristics. When the significance threshold is less than 0.3, Npsc and Nps drastically decrease, but the ratio (Nps/Npsc) rapidly increases. It can be considered that the selected PS and PSC with a significance less than 0.3 under the polarimetric stationarity hypothesis have polarimetric stationarity. When the significance is 0.3, the proportion of PS in the total pixels (1080000) is 3.69%, and the proportion of PS in the PSC is 14.78%. Therefore, the range of significance threshold is 0.1–0.3.

**Figure 6:**
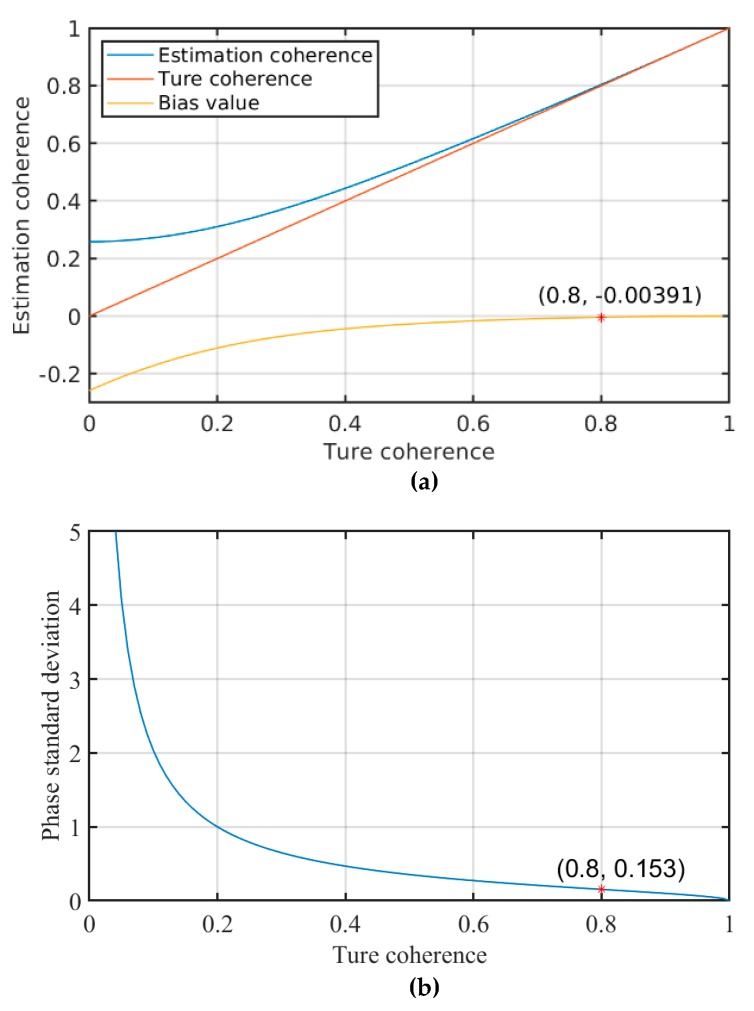
Statistical characteristics of temporal coherence estimation with different coherence value and 12 interferograms. (**a**) Estimated coherence, true coherence and the bias value; (**b**) variance of estimation coherence.

The temporal coherence is an important indicator for evaluating the phase quality of both PSC and PS. In order to evaluate the bias of coherence estimation, the paper makes a detailed analysis on the statistical characteristics of temporal coherence estimation, as shown in Figure 6. Touzi et al. illustrated the deviation between estimated coherence and true coherence for statistically independent samples [41]:

(13) $$d=\frac{\Gamma \left(L\right)\Gamma \left(\frac{3}{2}\right){\left(1-D^{2}\right)}^{L}}{\Gamma \left(L+\frac{1}{2}\right)}{\;}_{3}F_{2}\left(\frac{3}{2},L,L;L+\frac{1}{2},1;D^{2}\right)$$

where D is the true degree of coherence, d is the estimated coherence, Γ denotes the gamma function,${\;}_{3}F_{2}$ denotes the hypergeometric function, and L is the number of statistically independent samples. Then the phase standard deviation under different coherence was obtained [36]:

(14) $$\sigma_{\varphi }=\sqrt{\frac{1-{\left|D\right|}^{2}}{2L{\left|D\right|}^{2}}}$$

The following is to quantitatively analyze the estimated bias and the phase standard deviation condition of the temporal coherence with different coherence value and number of interferograms. Figure 6 shows that when the number of samples is 12, the deviation between estimated coherence and true coherence is very small in the high-coherence region (the main feature of PS). When the coherence is 0.8, the deviation of coherence is −0.00391, and the variance of estimated coherence is 0.0228, which shows that the estimated coherence can evaluate the performance of the proposed method.

In order to compare the effect of the PSOT on different polarizations, the thresholds are set to 0.1, 0.2 and 0.3 respectively to select the points in ESPO, HH, HV and VV. The PSC (PS) coherence distribution of different polarizations at the same threshold can reflect the performance of the ESPO.

**Figure 7:**
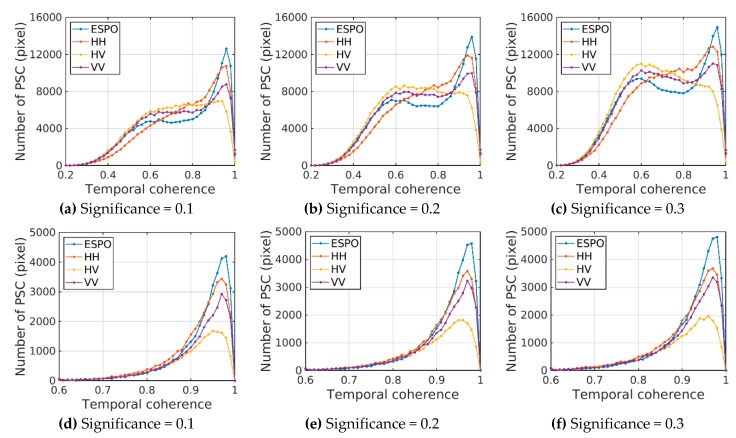
Coherence distribution is selected for different polarizations (ESPO, HH, HV, VV) under different significance thresholds; above is the coherence distribution of PSC, below is the coherence distribution of PS; (**a**,**d**) significance = 0.1; (**b**,**e**) significance = 0.2; (**c**,**f**) significance = 0.3.

Figure 7 shows that the temporal coherence of PSC identified by the PSOT method to the ESPO polarization is the lowest. For HH and VV polarizations, the PSOT selects more high-coherence pixels (> 0.7) than VV polarizations, and selects fewer low-coherence pixels (< 0.7). The ESPO selects less PSC in the coherence range of 0.6–0.85 than other polarizations, and selects the most PSC in the coherence range of greater than 0.85, which makes the distribution of PS more concentrated in the high-coherence region.

### 4.2. Comparison of Different PSC Selection Methods

In order to compare the difference between this method (PSOT+ESPO) and the traditional method, we compared the performance of amplitude dispersion index (ADI) in selecting PSC for different polarizations (ESPO, HH, HV, VV). The ADI and the PSOT describe the stability of scatterer from different angles, so the threshold value is not comparable. However, the purpose of different PSC selection methods is to select the "optimal" PSC set, so it is more reasonable to compare the distribution of time coherence in the same number of PSC sets. Based on PSOT, different significance thresholds (0.1, 0.2, 0.3) are used to select PSC, and then the same number of PSCs for different polarizations are selected by the ADI to compare. The following table shows the actual thresholds of PSCs selected by different methods.

**Figure 8:**
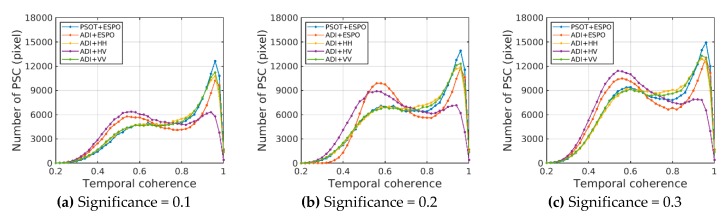
PSC temporal coherence distribution is selected for different methods under different significance thresholds; (**a**), significance = 0.1; (**b**), significance = 0.2; (**c**), significance = 0.3.

Figure 8 shows that the effect of the ADI+ESPO is only better than that of the ADI+HV, which shows that, with the ESPO method based on ADI index, it is easy to select low-coherence PSC in the aspect of selecting PSC by mistake. The traditional co-polar HH and VV can effectively select high-coherence PSC. The main reason is that the co-polar polarization contains most of the scattering energy of the ground objects. It is worth noting that when the PSOT is used to select PS, the best performance can be achieved by using the ESPO method to optimize polarization, which is more concentrated in the region with coherence greater than 0.9. This shows the effectiveness of the PSOT in PSC selection, and effectively makes up for the shortcomings of the ESPO method. Moreover, under different confidence thresholds, the PSOT has the same effect and good robustness.

**Figure 9:**
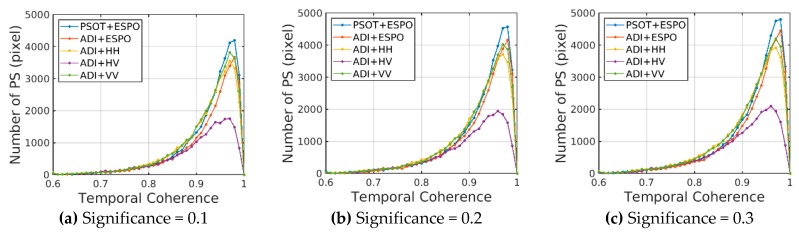
PS temporal coherence distribution is selected for different methods under different significance thresholds; (**a**) significance = 0.1; (**b**) significance = 0.2; (**c**) significance = 0.3.

To further compare the performance of different methods, it is necessary to compare the coherence distribution of PS. In this paper, the same PSI process is used to process the PSC obtained by different methods, and the coherence distribution of PS obtained by various methods (Figure 9).

After removing most of the low-coherence PSC, the coherence of PS is mainly distributed in the range above 0.8. The performance of the ADI+ESPO is equivalent to that of the ADI+HH/VV. This is because the ESPO method based on ADI is still the statistical information based on a single polarization, which is easy to greatly reduce the amplitude dispersion index of the scatterer (see the threshold value in Table 2). This will result in a larger proportion of points that are unstable being selected as PSC, so the amplitude dispersion index can not identify the PSCs of the ESPO very well. In this method, the quad-polarimetric information of the scatterer in time-series is considered to represent the stability of the scatterer, which avoids the limitation of a single measure. It can effectively make up for the shortcomings of the ADI+ESPO and select PSCs more effectively.

The performance of the ESPO has been discussed in [22,23,34]. In the experiment in this paper, we also found that the EPSO method can suppress the side lobe effect to a certain extent, and the texture of the surface coverage is clearer. We optimized the polarization of the quad-polarization image with an accuracy of 6°. To evaluate the effect of the ESPO, we compared it with three single polarization (HV and VH are equivalent) respectively. In order to compare the ability of different polarization to retain details of ground targets, representative buildings and parks were selected for analysis. The effects of different polarization will be analyzed in terms of intensity details and phase quality.

**Figure 10:**
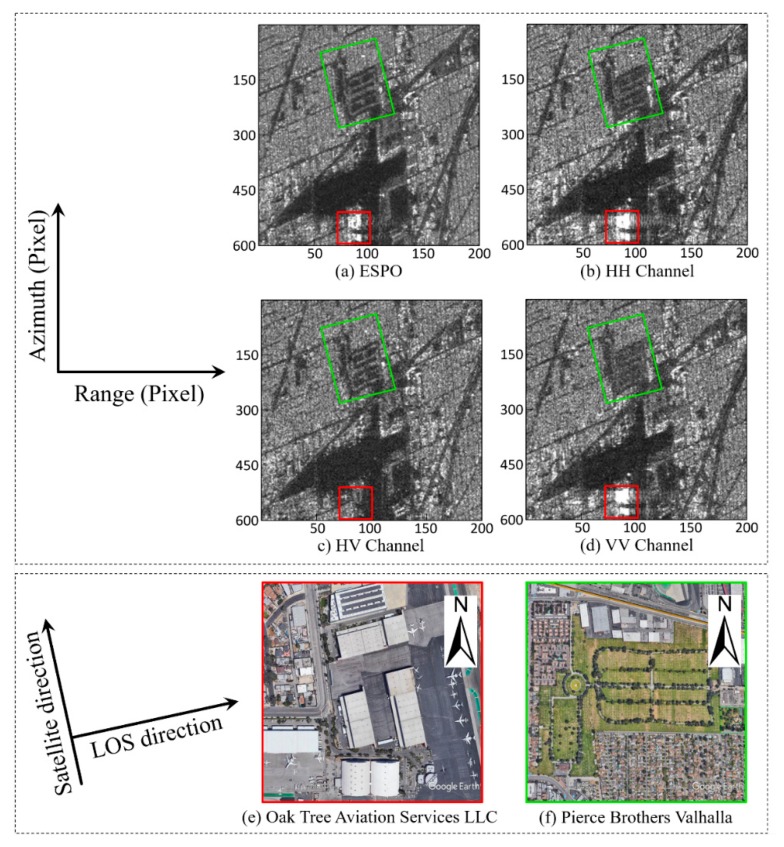
Intensity images of ESPO, HH, VV and HV polarizations, and Google Earth images of two ground objects; the red box is Oak Tree Aviation Services LLC and the green box is Pierce Brothers Valhalla. (**a**) ESPO; (**b**) HH; (**c**) HV; (**d**) VV; (**e**) Google Earth image of Oak Tree Aviation Services LLC; and (**f**) Google earth image of Pierce Brothers Valhalla. The strong scatterers in the red box show the effect of ESPO on sidelobe suppression, and the park in the green box shows the effect of ESPO on noise suppression and detail retention.

Figure 10a,b and d shows that ESPO inhibits the sidelobe effect to some extent. In Figure 10, the building with sidelobe is Oak Tree Aviation Services LLC, Burbank Glendale Pasadena (BUR) airport and the direction of the building is parallel to the LOS (line of sight) direction (Figure 10e), therefore the sidelobe effect is very serious in the co-polarized polarization (Figure 10b,d). However, the signal of BUR is weak in cross-polarized polarization (Figure 10c). ESPO not only suppressed the sidelobe effect, preserved the scattering information of the building, but also solved the problem that it was difficult to observe the building in the cross-polarized polarization. For Pierce Brothers Valhalla in Figure 10f, ESPO preserves scattering information of the park path with clear details and noise suppression. The noise suppression effect of ESPO is also well reflected in the odd scattering regions (airport runway, etc.).

## 5. Analysis of Deformation Results

**Figure 11:**
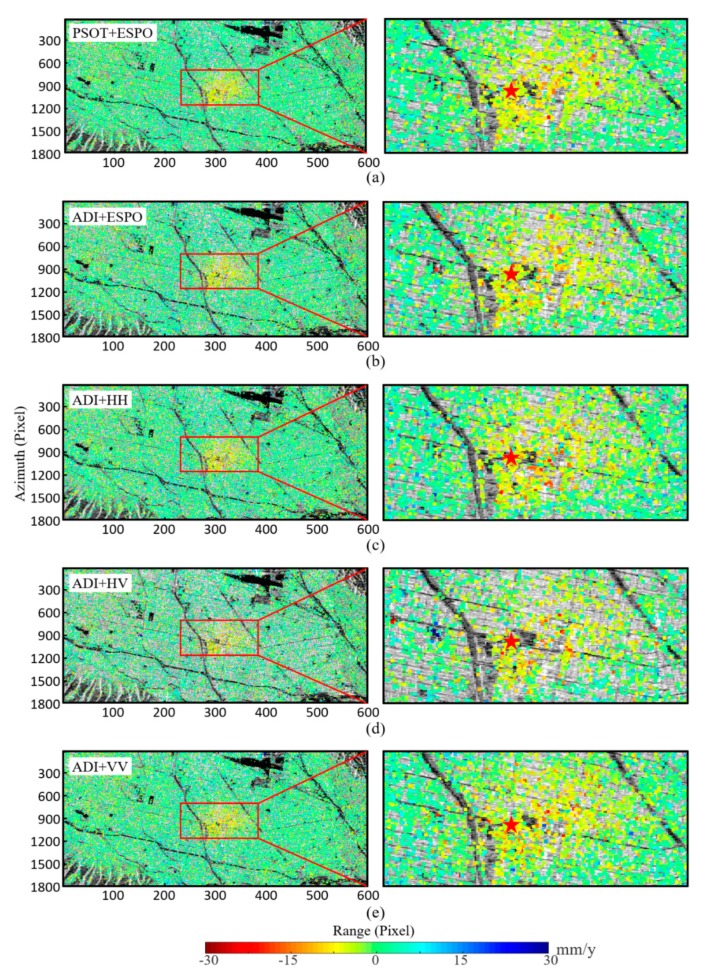
Mean deformation rate obtained by different methods (**a**) PSOT+ESPO; (**b**) ADI+ESPO; (**c**) ADI+HH; (**d**) ADI+HV; (**e**) ADI+VV; PSOT and ADI are the methods to identify PSC. the left shows the deformation results of the experimental area, the right is a zoomed-in view of deformation; the pentagram is position of the North Hollywood Station.

After verifying the performance of PSOT in Section 4, combining the constraint degree of significance threshold on the number of PSs, we select the median value (0.2) of the suggested significance threshold interval ([0.1 0.3]) for PSC selection. Then the ESPO method is carried out on the selected PSC, and the PS and time-series deformation is obtained by StaMPS (Figure 11a). In order to compare the effect of deformation monitored with different method, the ADI+ESPO (or HH, HV, VV) uses the same post-processing.

**Figure 12:**
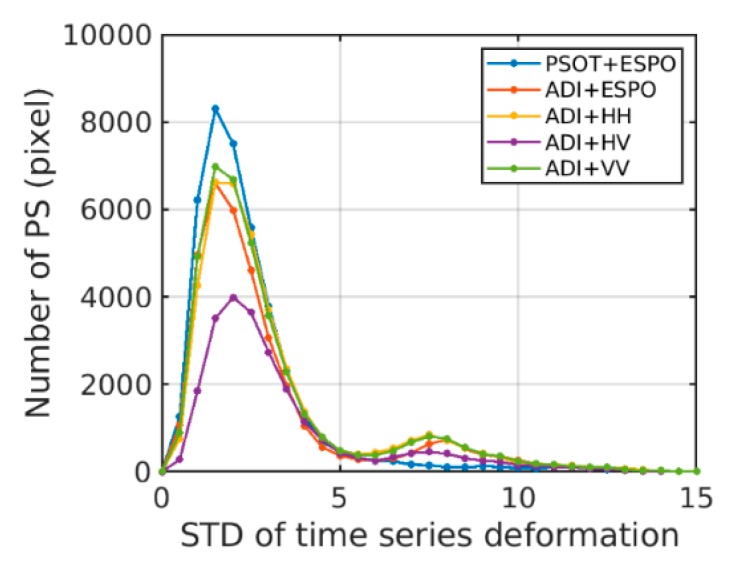
STD distribution of time-series deformation results obtained by different methods. STD is calculated by the residual time series after subtracting the linear component, units of STD is mm.

Figure 11a,d shows that the selected PS density of the PSOT+ESPO is higher than that of the ADI+HV, and there is less noise. Figure 11a–c,e looks similar, and the effect of deformation needs to be observed locally. It can be seen that Figure 11c,e has more noise pixels. The PSOT+ESPO and the ADI+ESPO have fewer noisy pixels. In order to quantitatively analyze the deformation results, we compared the standard deviation (STD) distribution of the time-series deformation of the five methods (Figure 12). PSs with larger STD indicate either larger errors (due to atmosphere or unwrapping errors) or deformation that is non-linear.

Figure 12 shows that the STD distribution of time-series deformation obtained by the PSOT+ESPO is significantly better than the other method. But the ratio of the ADI+ESPO in error points is higher than ADI+HH/VV, which also indicates that there is a problem of misselection. The PSOT method proposed in this paper can select the ESPO points more effectively, and the error points are far less than the ADI+ESPO/HH/VV. Specifically, in Table 3, we can see that only 2393 pixels of the deformation obtained by the PSOT+ESPO have STD greater than 5. For the specific deformation time-series, select the corresponding points of the pentagram in Figure 11 for analysis.

**Figure 13:**
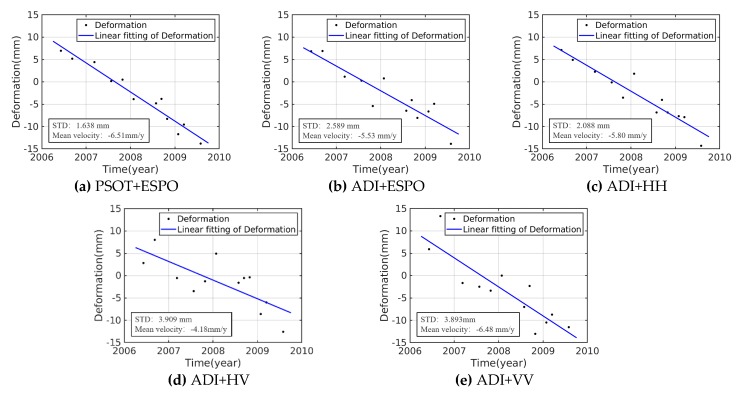
Time-series deformation of the pentagram in Figure 11; (**a**) PSOT+ESPO; (**b**) ADI+ESPO; (**c**) ADI+HH; (**d**) ADI+HV; (**e**) ADI+VV. STD is calculated by the residual time-series after subtracting the linear component.

Figure 13 shows that the time-series deformation of different methods. Assuming that the deformation is linear. Deformation rate estimation of ADI+HV is different from other methods, and its STD is the largest, so the reliability of this method is the lowest. Figure 13a shows that the time-series deformation with PSOT+ESPO has a higher linear fitting degree (STD = 1.638 mm) and its deformation rate estimation is similar to ADI+VV.

In order to compare the efficiency of different methods, the paper records the time consumption of different methods (Table 4). The ADI+HH/HV/VV methods do not require polarization optimization, so there is no ESPO time cost. Time consumption for PSI (StaMPS) includes coherence estimation, error estimation, phase unwinding, deformation estimation and so on. All the programs of these methods are executed under the condition of single-core processor without parallel processing.

The time consumption shown in the figure can be seen that the processing time for different methods to select the same number of PSC in PSI is equivalent. The PSOT+ESPO method proposed in this paper (the significance threshold is 0.2) is 79% less than the traditional ESPO for deformation monitoring. In addition, the calculation amount of the ESPO varies exponentially with accuracy. In this paper, we use accuracy of 6°. Reducing the accuracy of ESPO can also reduce the amount of calculation.

The deformation area monitored in this paper is about 3 km^2^ and the center is North Hollywood Station. The deformation is funnel-shaped, and the deformation rate is approximately linear. During the monitoring period, there was no major earthquake damage in the deformation area, and most of the buildings were built early. North Hollywood Station was also unable to cause the deformation of such a large area as 3 km^2^. Therefore, it is speculated that this is the inelastic deformation caused by groundwater exploitation.

## 6. Conclusions

The PSI is an important means for InSAR to monitor surface deformation. Improving the density and phase quality of PS are key problem in PSI. The ADI in the traditional single-pol PSI is difficult to describe the statistical characteristics of scatterers, so it is not robust when selecting PSC. In this paper, we propose a PSOT method to identify the polarimetric stationary scatterers as the PSCs. Experimental results show that the phase quality of PSCs identified by the PSOT+ESPO is higher than that by the ADI+ESPO method and traditional single-pol PSI. Through error analysis, the proposed PSOT+ESPO method can obtain the maximum number of PS, and the deformation estimation is more robust. Specifically, when the significance threshold is 0.2, 219006 PSCs were selected. After error analysis, 39620 PS remained, accounting for 3.67% of the total pixels (1080000).

Experiments show that the ESPO method can not only achieve the polarimetric optimization of the PSC interferometric phase, but also suppress the sidelobe of the strong scatterer effectively and make the details of the ground object clearer. The ESPO improves the coherence of the scatterers by optimizing the amplitude dispersion index, which will select some incorrect PSC when using the ADI method. The PSOT method based on the polarimetric SAR image can avoid the unsteadiness of PSC selected by the ESPO using ADI. Therefore, The PSOT combined with ESPO can identify PSC more accurately and improve the phase quality.

## Figures and Tables

**Table 1:** Temporal and spatial baselines of ALOS PALSAR-1 quad-polarimetric image.

Date	Perpendicular Baseline (m)	Temporal Baseline (Days)
20060608	−1129.9990	−322.00199
20060908	749.6921	−230.00109
20070311	1092.7781	−46.00006
20070426	0.0000	0.00000
20070727	1338.2859	91.99989
20071027	2264.7495	183.99958
20080127	2754.1354	275.99899
20080729	518.1668	459.99757
20080913	−1637.5801	505.99824
20081029	−1356.7558	551.99885
20090129	−655.3229	643.99985
20090316	−61.6506	690.00021
20090801	−204.9118	828.00092

**Table 2:** The threshold value of different PSC selection methods and the number of selected PSC.

Method	Threshold 1	Threshold 2	Threshold 3
PSOT+ESPO	0.1	0.2	0.3
ADI+ESPO	0.1680	0.1773	0.1851
ADI+HH	0.3628	0.3822	0.3985
ADI+VV	0.3750	0.3934	0.4088
ADI+HV	0.3636	0.3830	0.3993
Number of PSC	166081	219006	269638

**Table 3:** The mean intensity of each polarization and the polarization-optimized image.

Method	PSOT+ESPO	ADI+ESPO	ADI+HH	ADI+HV	ADI+VV
Number of PS	39620	35185	38408	23923	39015
Number of PS (STD > 5)	2393	5127	6263	3956	6113

**Table 4:** Calculation time of the proposed PSC selection and non-selection of PSC (CPU: AMD Ryzen 5 2600 Six-Core Processor × 12, Memory: 64G DDR4, Operation system: Ubuntu 16.04 LTS 64bit).

Time Consumption (h)	PSOT+ESPO	ADI+ESPO	ADI+HH	ADI+HV	ADI+VV
PSOT	0.080	—	—	—	—
ESPO	8.195	40.230	—	—	—
PSI(StaMPS)	0.155	0.154	0.173	0.140	0.171
Total	8.430	40.384	0.173	0.140	0.171
